# Think outside the pox

**DOI:** 10.1017/ash.2023.129

**Published:** 2023-04-14

**Authors:** Abbye W. Frederick, Anusha Govind, Laila M. Castellino

**Affiliations:** 1 UT Southwestern Medical Center, Division of Infectious Diseases and Geographic Medicine, Dallas, Texas; 2 Parkland Health, Dallas, Texas

Amid a worldwide outbreak of mpox (monkeypox), 847 infections were confirmed in Dallas County, Texas, between June and November 2022, with a peak in August.^1^ Yet in 2021, only 38 cases of confirmed and probable varicella were reported (Marc Williamson RN, email communication, October 2022).

In August 2022, a 35-year-old immunocompetent male was admitted to Parkland Health, with a 1-week history of rash on his arms, which spread diffusely across his face, torso, and genitals, a fever, nausea, and sore throat. He denied sick contacts, reported a single female sexual partner, and had immigrated from Mexico to the United States in 2021. He worked in construction, lived with his uncle and friend, and had no knowledge of childhood immunizations.

On examination, he was febrile with >1,000 discrete vesicular, pustular and umbilicated skin lesions in various stages, sparing the palms, soles, and oropharynx (Fig. 1). The patient had no lymphadenopathy; cardiac, respiratory, and neurological examination was unremarkable. The patient was suspected of having mpox and was placed under contact and airborne isolation precautions throughout hospitalization. Results of complete blood count, renal and hepatic function tests were normal. The human immunodeficiency virus (HIV) test result was negative.

**Fig. 1.**
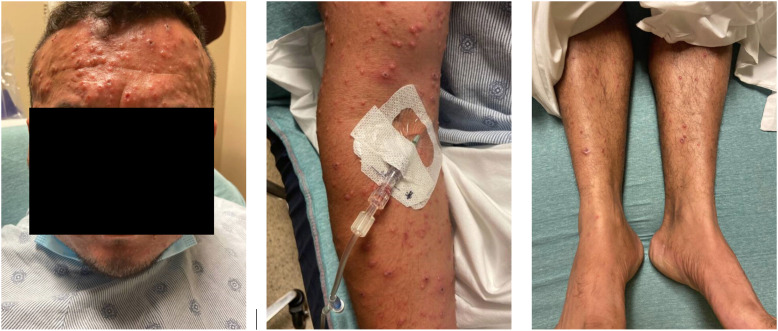
Skin lesions present on admission.

Although the differential diagnoses included disseminated herpes zoster and varicella, mpox was strongly suspected based on the epidemiology and Centers for Disease Control (CDC) case definition of a characteristic rash^2^ and empiric oral tecovirimat was started. However, orthopoxvirus polymerase chain reaction (PCR) testing of the lesions was negative, prompting testing for herpes and varicella viruses. After 6 days, lesions crusted and he was discharged home. A PCR assay of the lesions sent on hospital day 2 returned positive for varicella zoster virus (VZV). The case was reported to the public health department as primary varicella. The patient reported that his cousin whom he had spent time with, developed varicella and recovered. No additional cases were linked to this patient.

We diagnosed a case of primary varicella in an immunocompetent adult amid an mpox outbreak. Both viruses can be spread by direct contact, inhaled aerosols from vesicular fluid of skin lesions, and possibly through aerosolized infected respiratory secretions, leading to recommendations for contact and airborne precautions.^3^

The classic form of mpox described in Central and West Africa has an incubation period of 3–34 days, a prodrome of fever, headache, lymphadenopathy, and an eruption phase. Lesions spread in different stages evolving from macules, papules, vesicles, to pustules and can be umbilicated.^4^ Less commonly, lesions become ulcerated and necrotic. In the current outbreak of mpox, the disease has been transmitted primarily through sexual networks, leading to atypical presentations including asynchronous lesions clustered around the genital and anorectal region, with painful inguinal lymphadenopathy, pharyngitis, and proctitis.^4^

Primary varicella is classically a childhood illness, with vesicles starting on the chest and back, spreading centrifugally, associated with fevers, malaise, and headaches, with a higher incidence in spring and winter.^3,7^ The rash can be pleomorphic, progressing from macules and vesicles to scabs in different stages, while appearing umbilicated as they heal, resolving in 5–7 days.^3^ Among vaccinated patients, breakthrough varicella resolves more quickly, has fewer lesions, and presents with a maculopapular rash.^3^

Certain clinical features distinguish mpox from varicella. Lymphadenopathy is primarily seen in mpox and lesions often involve palms and soles. Varicella lesions are pleomorphic, in different stages of development,^3^ and generally spare the palms and soles. Difficulties in clinically differentiating the 2 diseases are well described in reports from the Democratic Republic of Congo where both diseases are endemic. Among 1,025 patients with suspected mpox, 383 patients demonstrated VZV only, not mpox. Among patients with varicella, 86% had lesions on their palms or soles, whereas 70% had lymphadenopathy, highlighting the difficulty in relying on clinical characteristics alone to distinguish the 2 diseases.^5^

In the 25 years since varicella vaccination was implemented in the United States, the incidence of varicella has decreased by 97% with only 5% of cases in those aged >10 years.^6^ Given this decline, healthcare professionals who have recently trained in the United States are less familiar with varicella’s clinical manifestations. Although the World Health Organization does not include varicella in its list of routinely recommended vaccines, it has been available in Mexico since 2000, yet is not widely offered. One-third of Latin Americans aged >10 years are susceptible to varicella, and in Mexico, 10% of cases occur in adults aged 25–44 years.^7^ Among immigrant populations, varicella should be considered in the differential diagnosis if the clinical context is appropriate. The CDC recommends clinicians test and update or revaccinate immigrants and refugees as appropriate, at the first health visit.^8^

Although the incidence of mpox has declined,^1^ it remains unclear whether it will be eradicated. Varicella epidemiology is also changing, with waning vaccine-induced immunity, and many populations, including immigrants, lacking immunization. Clinicians should consider the broad differential diagnosis, recognize the subtle clinical differences and the limitations of relying on epidemiological or clinical features alone, and test to establish the diagnosis in these settings. These actions, with prompt initiation of infection prevention and public health measures are key to limiting transmission.

## References

[1] Monkeypox. HHS-pages-one. Dallas County Health and Human Services website. https://www.dallascounty.org/departments/dchhs/monkeypox.php. Published June 28, 2022. Accessed December 2, 2022.

[2] Case definitions for use in the 2022 Mpox response. Centers for Disease Control and Prevention website. https://www.cdc.gov/poxvirus/monkeypox/clinicians/case-definition.html. Published October 26, 2022. Accessed January 5, 2023.

[3] Chickenpox (varicella) for healthcare professionals. Centers for Disease Control and Prevention website. https://www.cdc.gov/chickenpox/hcp/index.html. Published October 21, 2022. Accessed January 15, 2023.

[4] Gessain A , Nakoune E , Yazdanpanah Y. Monkeypox . N Engl J Med 2022;387:1783–1793. 36286263 10.1056/NEJMra2208860

[5] Leung J , McCollum AM , Radford K , et al. Varicella in Tshuapa Province, Democratic Republic of Congo, 2009–2014. Trop Med Int Health 2019;24:839–848. 31062445 10.1111/tmi.13243 PMC8786670

[6] Gershon AA , Breuer J , Cohen JI , et al. Varicella zoster virus infection. Nat Rev Dis Primer 2015;1:15016. 10.1038/nrdp.2015.16 PMC5381807 27188665

[7] Vergara-Castañeda A , Escobar-Gutiérrez A , Ruiz-Tovar K , et al. Epidemiology of varicella in Mexico. J Clin Virol 2012;55:51–57. 22750018 10.1016/j.jcv.2012.06.004

[8] Guidance for evaluating and updating immunizations during the domestic medical examination for newly arrived refugees. Centers for Disease Control and Prevention website. https://www.cdc.gov/immigrantrefugeehealth/guidelines/domestic/immunizations-guidelines.html. Published August 11, 2022. Accessed January 15, 2023.

